# Relationship between formulaic breast volume and risk of breast cancer based on linear measurements

**DOI:** 10.1186/s12885-020-07499-5

**Published:** 2020-10-12

**Authors:** Xiaoxia Li, Chunlan Zhou, Yanni Wu, Xiaohong Chen

**Affiliations:** 1 Department of Plastic and Cosmetic Surgery, Nanfang Hospital, Southern Medical University, 1838 Guangzhou North Road, Guangzhou, Guangdong 510515 P. R. China; 2 Department of Nursing, Nanfang Hospital, Southern Medical University, 1838 Guangzhou North Road, Guangzhou, Guangdong 510515 P. R. China; 3 Department of Thyroid Breast Surgery, the First Affiliated Hospital of Shantou University Medical College, 57 Changping Road, Shantou, Guangdong 515041 P. R. China

**Keywords:** Breast cancer, Breast volume, Propensity score matching, Linear measurement

## Abstract

**Background:**

Whether breast volume is a risk factor for breast cancer is controversial. This study aimed to evaluate whether a significant association between breast volume and risk of breast cancer, based on linear measurements, was present by applying propensity score matching (PSM).

**Methods:**

The study was designed as a hospital-based case-control study. Between March 2018 and May 2019, 208 cases and 340 controls were retrospectively reviewed. Information on menarche, smoking, feeding mode, oral contraceptives, reproductive history and family history was obtained through a structured questionnaire. Breast volume was calculated using a formula based on linear measurements of breast parameters. Cox regression and PSM were used to estimate odds ratios and 95% confidence intervals for breast cancer using risk factors adjusted for potential confounders.

**Results:**

There was a significant difference in breast volume between the two groups before propensity score matching (*P* = 0.014). Binary logistic regression showed that the risk of breast cancer was slightly higher in the case group with larger breast volumes than in the control group(*P* = 0.009, OR = 1.002, 95%*CI*:1.000 ~ 1.003). However, there was no significant statistical difference between the two groups using an independent sample *Mann-Whitney U* test (*P* = 0.438) or conditional logistic regression (*P* = 0.446).

**Conclusions:**

After PSM for potential confounding factors, there is no significant difference in breast volume estimated by BREAST-V formula between the case group and the control group. The risk of breast cancer may not be related to breast volume in Chinese women.

## Background

In recent years, breast cancer has become the most common cancer among women. In America, the number of new cases of breast cancer in women from 2011 to 2015 was 126.0 per 100,000 women per year, there were expected to be 266,120 new cases of female breast cancer, with an estimated 40,920 deaths from breast cancer in 2018 [1]. Up to 12.4% of women will be diagnosed with breast cancer. In China, the incidence of breast cancer has consistently ranked first among cancers in women. Studies have shown that menarche age [2], age at first pregnancy [3], feeding mode [4], family history of breast cancer [5], age [6], body mass index (BMI) [7], alcohol consumption [8], smoking [9], history of proliferative benign breast disease [10], oral contraceptives [11], abortion [12], breast density [13, 14], history of hyperthyroidism [15], and even night shift work [16], exercise [17], and diet [18] were related to the onset of breast cancer; regrettably no consensus has been reached [19, 20]. In short, breast volume has not been included in the traditional breast cancer risk list.

Hsieh et al. [21] found that premenopausal women who do not wear bras had half the risk of breast cancer of bra users (*P* approximately 0.09), possibly because they were likely to have smaller breasts. This also suggested a relationship between breast volume and the risk of breast cancer. However, in a population-based nested case-control study, Thurfjell et al. [22] found that small breast size was associated with increasing breast cancer risk, and they speculated that this may be related to breast density. Eriksson et al. [23] described the relationship between breast size and breast cancer risk from a genetic perspective, failing to define the relationship between the two. Therefore, more research is needed to clarify the relationship between breast volume and the risk of breast cancer.

To better provide a theoretical basis for breast cancer prevention, we conducted the present study to determine the association between breast volume and the risk of breast cancer. Longo’s [24] formula for calculating breast volume was used to calculate breast volume using linear measurement data.

## Methods

### Patients and procedures

This retrospective hospital-based case-control study was conducted to investigate the association between breast volume and the risk of breast cancer among women; it involved 208 cases and 340 controls from March 2018 to May 2019 from two hospitals in Guangdong Province, one of which was a tertiary hospital with nearly 3000 beds. Eligible participants were asked to complete a questionnaire under the supervision of trained interviewers; therefore, the loss of sample size due to lost interviews was not considered. The case group included women diagnosed with breast cancer through pathological examination, while the control group included women who underwent health screening examinations, including molybdenum target X-ray breast examinations, and women who had breast cancer within 1 month were excluded. The case group and control group were matched in a ratio of 1:1. To achieve a power of 85% and a two-tailed type I error rate of α = 0.05, each group required at least 181 patients. Considering the propensity score matching success rate, we collected more than this number of cases and more controls to obtain optimal match. The case group included adult women who had recently been diagnosed with breast cancer and were preparing for surgery or adjuvant chemotherapy, excluding those who were pregnant or breastfeeding or who had a history of breast cancer surgery, breast masses, breast augmentation, or communication difficulties. Healthy adult women who were examined at the hospital to ensure that they did not have breast cancer were enrolled in the control group, excluding those who were pregnant or breastfeeding or who had undergone breast augmentation, suspected breast cancer, or communication difficulties.

Information on sociodemographic characteristics, menarche age, alcohol consumption, smoking, history of proliferative benign breast disease, feeding mode, oral contraceptives, reproductive history, and family history of breast cancer was obtained through a structured questionnaire. The breast parameters of the two groups were measured and breast volume was calculated using a formula based on linear measurements of breast parameters [24].

### Exposure and covariate determination

The dependent variable for this study was cancer status (as a dichotomous variable with 1: breast cancer diagnosis; 0: nonbreast cancer diagnosis), and the only exposure factor was breast volume, which was calculated from linear measurements using the BREAST-V formula [24]. Breast volume was a continuous variable, and other variables, such as age, BMI, menarche age, age at first pregnancy, number of pregnancies, feeding mode, history of proliferative benign breast disease, history of oral contraceptives, smoking, alcohol consumption, history of hyperthyroidism, and family history of breast cancer were assessed at the baseline with the questionnaire.

### Collection of linear breast measurement data collection

All data in this study were collected during in-person interviews after consent was obtained from all study participants; bilateral breast data were collected. The measurer was single-blind to grouping. Anatomical distances included in the BREAST-V formula were the sternal notch-to-nipple distance, fold-to-nipple distance, and fold-to-fold projection distance when the measured person was in a standing position.

### Statistical analysis

This study was a case-control study. All data were entered by two people after verification and statistical processing was performed using SPSS 24.0. The normally distributed data were described as *M ± S*, and the independent sample t test was used for comparisons between two groups; the data with a skewed distribution were described by *M* (*P*_25_, *P*_75_), and the Mann-Whitney *U* test was used for comparisons between two groups. The count data were described by a ratio or composition ratio, and the chi-square test was used for comparisons between two groups. A level of *P* < 0.05 was used to indicate significance; all statistical tests were two-tailed.

### Propensity score matching

Propensity score matching was performed to control for potential confounders, and the match tolerance value was 0.005. The propensity scores were determined by using age, BMI, age at menarche, age at first pregnancy, number of pregnancies, feeding mode, history of proliferative benign breast disease, history of oral contraceptives, smoking, alcohol consumption, history of hyperthyroidism, and family history of breast cancer. The propensity value calculated according to the logistic regression was matched according to the 1:1 nearest neighbor matching method, and then the two matched groups were regarded as independent groups. The baseline data were statistically analyzed before and after matching. Binary logistic regression analysis was used before PSM, while conditional logistic regression analysis was performed with the help of a Cox regression model in SPSS 24.0 to evaluate the effect of breast size on the risk of breast cancer after matching. A virtual survival time was recorded for each row before and after matching. Survival time was regarded as a time variable, outcome was regarded as a status variable, and the remaining variables were regarded as covariates. The default “case group” had a short survival time, and the “control group” had a long survival time. The odds ratio (OR) for breast cancer was calculated in the highest vs lowest quartile of breast volume as the ratio between the observed prevalences, and it was expressed with a 95% confidence interval.

## Results

### Data and procedures

A total of 208 women were included in the case group, and 340 women were included in the control group. PSM was performed with SPSS 24.0, and 185 women were successfully matched after balancing the confounding factors of the two groups of patients.

The measured bilateral breast data were averaged, and the breast volume was calculated using the BREAST-V formula. Breast volume was regarded as a continuous variable and the only dependent variable was whether breast cancer was present.

### Comparison of baseline data between the two groups before matching

The measurement data included in this study were not normally distributed. The Mann-Whitney *U* test was used for comparisons between groups. The results showed that, except for age (*P* = 0.668) and BMI (*P* = 0.211), the differences in other indicators were statistically significant (menarche age, *P* < 0.001; age at first pregnancy, *P* = 0.012; number of pregnancies, *P* = 0.045). The menarche age in the case group was earlier than that in the control group, the age at first pregnancy was later than that of the control group, and the number of pregnancies was less than that of the control group. There were no significant differences in the count data between the groups using the chi-square test (feeding mode, *P* = 0.554; history of proliferative benign breast disease, *P* = 0.321; history of oral contraceptives, *P* = 0.932; smoking, *P* = 0.201; alcohol consumption, *P* = 0.121; history of hyperthyroidism, *P* = 0.589; family history of breast cancer, *P* = 0.196) (Table 1).

**Table 1:** Comparison of clinical characteristics before matching data between the two groups

Covariates	Control group	Case group	Test statistics	***P*** value
Age	50.4 (45.0, 54.0)	50.7 (45.0, 57.0)	*U* = 34,589.00	0.668
BMI	23.48 (21.10, 25.26)	23.72 (21.23, 26.04)	*U* = 33,110.50	0.211
Menarche age	14.9 (13.0, 16.0)	14.3 (13.0, 15.0)	*U* = 29,130.00	< 0.001
Age at first pregnancy	23.7 (21.0, 26.0)	24.3 (22.0, 26.0)	*U* = 30,871.50	0.012
Number of pregnancies	3.0 (2.0, 4.0)	3.0 (2.0, 4.0)	*U* = 31,835.50	0.045
Feeding mode			χ2 = 0.350	0.554
Breastfeeding	316 (92.9%)	196 (94.2%)		
Non-breastfeeding	24 (7.1%)	12 (5.8%)		
Proliferative benign breast disease			χ2 = 0.984	0.321
Yes	107 (31.5%)	74 (35.6%)		
No	233 (68.5%)	134 (64.4%)		
Oral contraceptives			χ2 = 0.009	0.923
Yes	27 (7.9%)	17 (8.2%)		
No	313 (92.1%)	191 (91.8%)		
Smoking			χ2 = 1.638	0.201
Yes	0	1 (0.5%)		
No	340 (100%)	207 (99.5%)		
Alcohol consumption			χ2 = 4.230	0.121
No risk	325 (95.6%)	193 (92.8%)		
Low risk	15 (4.4%)	13 (6.3%)		
High risk	0	2 (1.0%)		
History of hyperthyroidism			χ2 = 0.292	0.589
Yes	9 (2.6%)	4 (1.9%)		
No	331 (97.4%)	204 (98.1%)		
Family history			χ2 = 1.673	0.196
Yes	8 (2.4%)	9 (4.3%)		
No	332 (97.6%)	199 (95.7%)		

### Comparison of baseline data between the two groups after matching

There were no significant differences using the Mann-Whitney *U* test (*P* range of 0.484 to 0.983), chi-square test or the Fisher’s exact test between the two groups after PSM. The baseline data between the groups reached equilibrium (Table 2).

**Table 2:** Comparison of clinical characteristics after matching data between the two groups

Covariates	Control group	Case group	Test statistics	***P*** value
Age	49.9 (45.0, 54.0)	50.5 (44.0, 57.0)	*U* = 16,454.00	0.522
BMI	23.83 (21.24, 25.42)	23.65 (21.16, 26.04)	*U* = 17,032.00	0.938
Menarche age	14.4 (13.0, 15.0)	14.4 (13.0, 15.0)	*U* = 16,409.00	0.484
Age at first pregnancy	24.3 (21.0, 26.0)	24.1 (22.0, 26.0)	*U* = 17,101.50	0.991
Number of pregnancies	3.1 (2.0, 4.0)	3.1 (2.0, 4.0)	*U* = 17,090.50	0.983
Feeding mode			–	1
Breastfeeding	175 (94.6%)	175 (94.6%)		
Non-breastfeeding	10 (5.4%)	10 (5.4%)		
Proliferative benign breast disease			χ2 = 0.106	0.745
Yes	68 (36.8%)	65 (35.1%)		
No	117 (63.2%)	120 (64.9%)		
Oral contraceptives			χ2 = 0.492	0.483
Yes	20 (10.8%)	16 (8.6%)		
No	165 (89.2%)	169 (91.4%)		
Smoking			–	1
Yes	0 (0)	0 (0)		
No	185 (100.0%)	185 (100.0%)		
Alcohol consumption			χ2 = 1.387	0.239
No risk	178 (96.2%)	173 (93.5%)		
Low risk	7 (3.8%)	12 (6.5%)		
High risk	0	0		
History of hyperthyroidism	1 (0.5%)	4 (2.2%)	χ2 = 1.825	0.177
Yes	184 (99.5%)	181 (97.8%)		
No				
Family history			χ2 = 1.378	0.240
Yes	4 (2.2%)	8 (4.3%)		
No	181 (97.8%)	177 (95.7%)		

### Relationship between breast volume and the risk of breast cancer

The breast volume data before matching were normally distributed (*P* = 0.200 in the control group; *P* = 0.200 in the case group), and the variance was not uniform (*P* < 0.001). The Mann-Whitney *U* test using two independent samples showed a significant difference (*P* = 0.014) (Table 3). Binary logistic regression analysis of breast volume showed that the risk of breast cancer was slightly higher in the case group than in the control group (*P* = 0.009, *OR* = 1.002, 95% *CI*: 1.000 ~ 1.003). The odds ratio between the highest and the lowest groups based on quartile groups was 1.515; however, this result was not statistically significant (*P* = 0.089) (Table 4) .

**Table 3:** Comparison of breast volume before PSM

Group	cases	Mean Rank	Test statistics	***P*** value
Control group	340	261.44	*U* = 30,919.000	0.014
Case group	208	295.85

**Table 4:** Effect of 1 cm^3^ increase in breast volume after PSM on breast cancer risk and odds ratio between the highest and the lowest groups based on quartile groups

	B	S.E.	Wald	df	Sig.	Exp (B)	95% CI for Exp (B)	
							Lower	Upper
BREAST-V	0.002	0.001	6.728	1	0.009	1.002	1.000	1.003
BREAST-V(1)^a^	−0.502	0.258	3.796	1	0.051	0.605	0.366	1.003
BREAST-V(2)^b^	−0.209	0.251	0.690	1	0.406	0.812	0.496	1.328
BREAST-V(3)^c^	0.415	0.244	2.885	1	0.089	1.515	0.938	2.446

Divide the breast volume into quartile groups: ^a^Odds ratio between the second and the lowest group based on quartile groups; ^b^Odds ratio between the third and the lowest group based on quartile groups; ^c^Odds ratio between the highest and the lowest group based on quartile groups.

After PSM, breast volume did not have a normal distribution. There was no significant difference between the two groups using an independent sample Mann-Whitney *U* test (*P* = 0.438) (Table 5) or conditional logistic regression (*P* = 0.446) (Table 6).

**Table 5:** Comparison of breast volume after PSM

Group	cases	Mean Rank	Test statistics	***P*** value
Control group	185	181.19	*U* = 16,315.50	0.438
Case group	185	189.81

**Table 6:** Effect of 1 cm^3^ increase in breast volume after PSM on breast cancer risk

Covariates	Residual Chi Square	df	Sig.
Age	0.471	1	0.492
BMI	0.302	1	0.583
Menarche age	0.206	1	0.650
Age at first pregnancy	0.214	1	0.644
Number of pregnancies	0.011	1	0.918
Feeding mode	< 0.001	1	1.000
Proliferative benign breast disease	0.111	1	0.739
Oral contraceptives	0.571	1	0.450
Smoking	–	0	–
Alcohol consumption	1.316	1	0.251
History of hyperthyroidism	1.800	1	0.180
Family history	1.600	1	0.206
BREAST-V	0.581	1	0.446

## Discussion

A Chinese study showed that abortion does not increase the risk of breast cancer, and the latest meta-analysis did not find a relationship between abortion and breast cancer risk [12]. A study by Ilic et al. [25] suggested that even short pregnancies ending in abortion protect against breast cancer. Therefore, the covariates in this study included only the number of pregnancies instead of the number of deliveries.

A pooled analysis of 6 prospective cohort studies [26] indicated that women consuming 30–60 g/d of alcohol had a 41% higher risk of invasive breast cancer than nondrinkers; women consuming 60 g/d or more of alcohol had a 31% higher risk of invasive breast cancer; and women consuming less than 30 g/d of alcohol had at most a 16% higher risk of invasive breast cancer. Another study confirmed this interesting association [27]. Therefore, < 30 g/d of alcohol is defined as low-risk alcohol consumption; 30–60 g/d of alcohol is defined as high-risk alcohol consumption; and ≥ 60 g/d of alcohol is defined as medium-risk alcohol consumption.

Egan et al. [28] concluded that breast size is a positive predictor of postmenopausal breast cancer limited in those who were especially lean as young women from a population-based case-control study of women aged 50 to 79 years. Williams et al. [29] deemed baseline bra cup size to be the strongest predictor of breast cancer mortality. A prospective study by Kusano et al. suggested that for women with a BMI below 25 kg/m^2^, those with a bra cup size of “D or larger” had a significantly higher incidence of breast cancer than women who reported “A or smaller” (covariate adjusted *HR* = 1.80; 95% *CI* = 1.13–2.88; *p* trend = 0.01), although this association was limited to leaner women [30]. Some experts pointed out the shortcomings of this study, and they thought that the use of cup size alone without taking rib cage circumference into account was not rigorous [31]. In addition, cup size labeling was not standardized and different brands of brassieres differ in their labeling of cup size for the same breast volume. Breast size as measured by self-reported bra cup size was the biggest drawback of these studies.

For the measurement of breast volume, the gold standard is the water displacement method [32]. Apart from this, there are still no accepted standard methods. Three-dimensional ultrasound (3-D US) [32] is relatively similar to the water displacement method, but it is expensive and requires professional cooperation. 3D scanning is a new and more advanced method [33]. However, for women with larger breast volumes, 3D scanning is not accurate [34], and the technical and cost requirements are higher. Choppin et al. [35] considered magnetic resonance imaging (MRI) scans to have the highest accuracy after comparing 3D scanning, mammography, MRI, CT, model casting and other methods. Itsukage et al. [36] also believed that MRI is more accurate for measuring breast volume.However, it requires data analysis software and is more expensive. The BREAST-V formula used in this study is the first unified, effective and reliable breast volume prediction formula; it was designed by Longo [24]. It is the most common method used by the researchers’ unit and can be used to assess the volume of breasts of different sizes and is easy to operate without additional requirements for the measurement technique. The data are subjectively less affected, and it is more accurate for measuring breast volume than other methods.

In the present study, there was no significant difference in age between the case group and the control group before PSM. The reason may be that we excluded the lower age population in the process of collecting the case group data, and we found that the age of the physically examined population ranged from 40 to 70, which is similar to the high-risk age range for breast cancer onset. The reason for the lack of a significant difference in BMI between the two groups may be due to an insufficient sample size, in addition to not having ruled out the existence of Berkson bias. The prevalence of cigarette smoking among women in China is quite low compared to that of males, and our observations were characteristic of smoking in China. Therefore, the recruited participants seem to be a group of very healthy persons.

The advantage of this study is that our breast volume data were obtained using a simple and convenient method based on linear measurements of the breast; on the other hand, PSM was used to balance potential confounding between the case group and control group to ensure the comparability of the two groups to a certain extent. Compared with overfitting in traditional multivariate regression, overfitting in this study was reduced to some degree since the propensity score was applied to all the selected patients and finally matched according to the tendency score. In terms of the sample size, given the problem of unsuccessful matching, the control group had more data collected than the case group, which guaranteed the matching success rate to some extent.

The limitations of this study are as follows. First, there are inherent biases from this study. It is acknowledged that selection bias will have a strong effect on representativeness and prevalence estimates. To reduce this risk, only patients with incident breast cancer were chosen, while controls were recruited from among healthy persons instead of from among patients’ visitors to reduce confounding by family history. Second, recall bias is a well-known source of systematic error and may lead to spurious associations between exposures and the outcome of interest. In this study, recently diagnosed cases were included, and direct interviews of both the case and control groups were performed by the same trained interviewers to reduce recall bias and improve the accuracy of the information obtained. Third, young women who are breastfeeding have differences in breast volume from those who are not, and older women have reduced breast volume due to fat reduction; in addition, breast size data could not be obtained for patients who had undergone a double mastectomy. Therefore, although this study used PSM, imbalance cannot be completely eliminated, which is one of the shortcomings of this research. Finally, although the hospitals selected have a wide range of radiation, they are limited, and our findings cannot be generalized to the entire Chinese population because all participants were recruited from the same province area.

Although breast density is one of the risk factors for breast cancer [13, 14], regrettably, in the case of pathological examination-confirmed breast cancer, for ethical considerations, we cannot require all patients to undergo X-ray examinations to measure breast density. Therefore, we were unable to obtain breast density data for all participants. Fortunately, we obtained breast density data for some subjects and will focus on expanding the sample size in future studies. In addition, we have not been able to compare the BREAST-V formula with the gold standard for breast measurement, so we cannot judge its accuracy, although other studies have reported the accuracy of the BREAST-V formula. And there are big differences between different breast volume measurement methods. Compared with other methods, we don’t know more about the advantages and disadvantages of the BREAST-V formula.

## Conclusions

In this case-control study, we found a significant difference in breast volume between the two group. Regrettably, this difference disappeared after PSM. However, the effect of breast volume on the risk of breast cancer can not be ignored. It is suggested that subsequent research can be carried out with multiple centers and large sample sizes.

## Supplementary information

**Additional file 1.** Supplementary file 1: Table S1. Questionnaire.

## Data Availability

The datasets used and/or analysed during the current study are available from the corresponding author on reasonable request.

## Abbreviations

PSM: Propensity score matching
BMI: Body mass index
OR: Odds ratio
MRI: Magnetic resonance imaging
