# Supplementary material for: Relationship between formulaic breast volume and risk of breast cancer based on linear measurements
Source: BMC Cancer. 2020 Oct 12;20:989. doi: 10.1186/s12885-020-07499-5 (PMC7552486; doi:10.1186/s12885-020-07499-5)
Supplement: Supplementary file 1 — Additional file 1. Supplementary file 1: Table S1. Questionnaire. [file 12885_2020_7499_MOESM1_ESM.docx]

[Supplementary file 1: Table S1. Questionnaire.](https://www.ncbi.nlm.nih.gov/pmc/articles/PMC6833132/bin/12885_2019_6218_MOESM1_ESM.docx)

| Entry | Situation |
| --- | --- |
| age |  |
| height |  |
| body weight |  |
| menarche age |  |
| age at first pregnancy |  |
| number of pregnancies |  |
| feeding mode |  |
| proliferative benign breast disease |  |
| Are there oral contraceptives? If yes, please specify the number of years |  |
| Smoking：please indicate the frequency |  |
| alcohol consumption：please indicate the frequency |  |
| history of hyperthyroidism |  |
| family history of breast cancer |  |
| breast parameters (cm): SN:sternal notch-to-nipple distance; FFp： fold-to-nipple distance； FN： fold-to-fold projection distance |  |
